# Comparative Expenditure of Hospitals

**Published:** 1888-04-14

**Authors:** 


### Comparative Expenditure of Hospitals.

We are indebted to the authorities for the following interesting report of the committee appointed to consider the relative expenditure of a Northern Hospital compared with others in the neighbourhood. It is dated November, 1887, and is as follows :—

After visiting several hospitals mentioned in the statistical report compiled by the secretary, the committee have come to the following conclusions :—

Firstly.—That the staff is in every department larger than is necessary.

Secondly.—That a considerable saving might be effected by having a fixed scale of dietary, and in various reforms connected with the purchase of butcher's meat, the mode of cooking, and the serving up of dinners.

Thirdly.—That the Honorary Medical Staff should be requested to undertake the duty, as far as possible, of prescribing for the out-patients as well as for those in the Hospital.

Fourthly.—That recommendation forms for in-patients be discontinued.

The resolution of the board under which this committee was appointed forbids their entering upon the question of the resident medical staff; they therefore limit the proposed reductions to the household and the nursing departments. As regards the first, they propose that one laundry maid should be dispensed with, and that the kitchen maid should assist in the laundry a portion of two or three days in the week. The committee also consider a head nurse unnecessary, as the matron should occupy that position and take the complete charge and oversight of the nursing department. This is the rule in each hospital which has been visited except one, and if this recommendation is carried into effect there would then be for actual day duty three nurses (leaving out the matron and night nurse), which would average one nurse for each seven beds. Applying the same rule to eight other hospitals of not more than forty beds each, the committee find that this duty is not excessive, for the average is one for each 7·87 beds for day duty. It must be remembered that many patients are not confined to bed, and that some are consequently able to assist other patients in little matters.

The second conclusion connects itself principally with the dinners served in the establishment. At present there are about five separate joints cooked, consisting of ribs and sirloin of beef and legs of mutton; two are for the wards, one for the nurses, one for the servants, and one for the doctors. The matron, we believe, also dines separately, but has her dinner cut from one of the other joints. This division causes considerable waste and extra trouble; it is therefore suggested that as often as possible only one large joint should be cooked, from which should be first cut off in the kitchen by the matron, or cook under her personal superintendence, the dinners for the patients, which should be placed in tin boxes, warmed, and then enclosed in a larger case of the same material, and so carried into the ward kitchens, there to be transferred to the patients' plates. Then the joint hould be put again into the oven, and, if large enough, served in turns to each of the different tables.

They suggest also that the matron should dine either with the doctors or nurses; if with the former, that the present committee-room should be used by them as a dining-room, which would keep their own apartments pleasanter.

The committee also recommend the following scale and routine of dietary for

Ordinary Diet, to be called "O."

Breakfast, 7.30.—Tea with milk, sugar, and bread and butter *ad libitum*.

Dinner, 12.—For men: 5 ozs. cooked meat, 8 ozs. potatoes, bread and rice, barley or bread pudding; for women and children 10 to 15 years old: 4 ozs. cooked meat, 8 ozs. potatoes, bread and rice, barley or bread pudding; for children under 10 years old: 3 ozs. cooked meat, 8 ozs. potatoes, bread and rice, barley or bread pudding.

Tea, 4.30.—Tea, with milk, sugar, and bread and butter *ad libitum*.

Supper, 7.30.—Half a pint of milk and bread, or rice milk.

Weekly Routine of Dinners.

Sunday.—Roast leg of mutton.

Monday.—Potato hash, Irish stew, or hot pot, prepared from remains of cold meat, and if required from neck of mutton or beef.

Tuesday.—Roast round of beef.

Wednesday.—Boiled neck of mutton.

Thursday.—Potato hash, Irish stew, or hot pot, as on Monday.

Friday.—Soup and potato pie, or fish.

Saturday.—Round of beef (roast or boiled).

For "low diet" the same as "ordinary diet," except dinner, for which a pint of broth or rice milk may be substituted instead of meat and potatoes, and milk may also be given instead of tea if specified by the medical officer in charge of the case.

For "special diets" the committee recommend that the honorary medical officers should be asked to prepare a scale of diets suitable for different cases, with quantities of each per day. It is also suggested that each honorary medical officer should fill up a special order for these, and especially for all stimulants prescribed, and that a separate record should be kept of the quantities so given, as is done in most hospitals.

The third point concerns the duties of the honorary medical staff, and the committee find that in all other hospitals the honorary surgeons not only attend to the hospital patients, but see and prescribe for all out-patients who are able to attend at the dispensary. As this duty would involve increased time to be placed at the service of the institution, the committee recommend that their number should be increased, one of the present staff being made into the consulting physician, and that there should be three new honorary medical officers elected on the surgical staff, which would then consist of six, and that each of them should take a day in every week to visit the hospital and dispensary, and there prescribe for the out and in-patients, and also visit such home patients as the resident medical officers consider could be better treated in the hospital.

This brings us, fourthly, to the question of recommendations, and the committee feel satisfied that the medical officers are better judges of who are fit and proper persons to become in-patients than subscribers generally. They suggest therefore that the recommendation forms given to subscribers be altogether for out-patients, but of two kinds, one to be called "out" and the other "home," and that an annual subscriber of a guinea should receive four "out" or two "home" forms, one "home" being changeable for two "out" forms. Any subscriber would be able to inform himself as to whether a patient could walk to the dispensary or hospital, and for such as were unable he would give a "home" recommend, and to others an "out." The medical staff would then, from both these classes, select such cases as in their opinion would be better attended to in the hospital. This is the plan adopted by many hospitals, amongst others by Salford, Dewsbury, Wakefield, Stockport, and Leeds, and in all these places it is found to work well. It is believed that if this change be made the number of in-patients would increase naturally, and the subscribers would know that all suitable cases would receive the treatment most applicable and beneficial to the respective cases. When a case is certified suitable for "in" treatment, it might be required (if the board thinks it desirable) that an extra "home" recommend should be obtained.

The cost of drugs is still larger than in other hospitals, and the committee recommend that, if possible, a contract should be made with a chemist in the town, who would supply in small quantities, as required, at so much per cent. off "a published list."

The committee believe that the reforms thus indicated, if carried out under the superintendence of the various committees, would result in a greatly increased economy, and at the same time add to the efficiency of the institutions. But they have another recommendation to make which at first sight may appear likely to augment the working expenses. This is the addition of a non-resident clerk, whose duties should be to keep the books on such a system that the cost of any article or department could at any time be seen and compared, to canvass for and collect subscriptions, and to send out the Hospital Saturday and Sunday Reports, and if necessary to address shop meetings at mills, etc., before or during the times of collection, to examine or test all goods sent in by tradesmen, and generally to see the orders of the board and sub-committees carried out. Ever since the opening of the hospital the treasurer and the secretary especially have devoted a considerable amount of time to the clerical work necessary, and other members have also assisted, particularly in the Saturday and Sunday Funds collections; besides this assistance, which has been rendered gratuitously, the Board has paid about £25 for book-keeping and collecting subscriptions. If all this work were placed in the hands of the clerk the additional expense would probably not be more than £40 or £50 per annum, and we think the supervision exercised would, if a suitable person were selected, result, not in an increased, but a considerably decreased expenditure. In visiting various hospitals, we find that an officer of this kind is generally employed, and the advantages gained are considered to be well worth the small additional outlay, which in our case may easily be recouped by extra subscriptions and decreased expenditure in several departments.

We are asked to express an opinion in this case, and especially as to the sufficiency and suitableness of the dietary. As to the general circumstances of the report, we cannot but express our approbation of the energy and thoroughness with which the committee have conducted their inquiries, and the almost uniform wisdom of their conclusions. With regard to the dietary, the only criticism that suggests itself is that it does not err on the side of liberality. This is particularly evident in the case of supper, which strikes us as being quite insufficient. It must always be remembered that hospital patients are in no sense to be looked upon as paupers. They are in hospital for the purpose of being restored to health; and those measures which tend most promptly to their restriction are always in the long run the most economical. A liberal diet for every patient who can digest it is always to be preferred to a meagre one.

